# Research on a General-Type Hydraulic Valve Leakage Diagnosis Method Based on CLAF-MTL Feature Deep Integration

**DOI:** 10.3390/s26030821

**Published:** 2026-01-26

**Authors:** Chengbiao Tong, Yu Xiong, Xinming Xu, Yihua Wu

**Affiliations:** 1 College of Electrical and Mechanical Engineering, Hunan Agricultural University, Changsha 410128, China; cbtong@hunau.edu.cn (C.T.); 895916397@stu.hunau.edu.cn (Y.X.); yihuawu@hunau.edu.cn (Y.W.); 2 Intelligent Agricultural Machinery Equipment Hunan Key Laboratory, Changsha 410128, China

**Keywords:** valve internal leakage, LSTM, multi-task learning, fault diagnosis, multi-source signal fusion

## Abstract

As control and execution components within hydraulic systems, hydraulic valves are critical to system efficiency and operational safety. However, existing research primarily focuses on specific valve designs, exhibiting limitations in versatility and task coordination that constrain their comprehensive diagnostic capabilities. To address these issues, this paper innovatively proposes a multi-modal feature deep fusion multi-task prediction (CLAF-MTL) model. This model employs a core architecture based on the CNN-LSTM-Additive Attention module and a fully connected network (FCN) for multi-domain features, while simultaneously embedding a multi-task learning mechanism. It resolves the multi-task prediction challenge of leakage rate regression and fault type classification, significantly enhancing diagnostic efficiency and practicality. This model innovatively designs a complementary fusion mechanism of “deep auto-features + multi-domain features” overcoming the limitations of single-modality representation. It integrates leakage rate regression and fault type classification into a unified modeling framework, dynamically optimizing dual-task weights via the MGDA-UB algorithm to achieve bidirectional complementarity between tasks. Experimental results demonstrate that the proposed method achieves an R^2^ of 0.9784 for leakage rate prediction and a fault type identification accuracy of 92.23% on the test set. Compared to traditional approaches, this method is the first to simultaneously address the challenge of accurately predicting both leakage rate and fault type. It exhibits superior robustness and applicability across generic valve scenarios, providing an effective solution for intelligent monitoring of valve leakage faults in hydraulic systems.

## 1. Introduction

As a critical control component in hydraulic systems, the operational status of hydraulic valves directly impacts the reliability and safety of the entire system. Valve failures such as leakage and sticking not only result in fluid waste and reduced system efficiency but may also trigger serious production safety incidents. Therefore, research on pattern recognition and quantitative prediction of valve leakage faults holds significant engineering application value.

In the field of valve leakage detection methodology, Shan et al. [1] conducted systematic investigations into testing methods for hydraulic valve leakage rates, establishing a correlation model between leakage volume and sealing surface wear. Guo et al. [2] proposed intelligent identification of one-way valve leakage patterns using deep convolutional neural networks, offering novel insights into the interpretability of deep learning models for valve fault diagnosis. Xiong et al. [3] addressed the challenge of detecting minute internal leakage in check valves by proposing an EEMD-PSO-SVM pattern recognition approach, achieving a detection accuracy of 94% for such subtle leaks. This provides critical technological support for early warning systems targeting minor leaks in hydraulic systems. Zhu et al. [4] conducted an in-depth exploration of valve internal leakage identification and leakage rate quantification techniques, proposing a multi-feature fusion-based identification criterion that advanced valve internal leakage diagnosis from qualitative identification to quantitative analysis. Li et al. [5] proposed a method for identifying internal leakage in spring-loaded full-lift safety valves based on an improved CNN. By acquiring acoustic emission signals via high-frequency FPGA and integrating BiGRU with a selective kernel attention module (SKAM) to enhance temporal and multi-scale feature capture, they achieved an average recognition accuracy of 99.7%. Their approach to signal processing and feature enhancement for specific valve types provides a refined reference for applying deep learning in valve fault diagnosis.

With the rapid advancement of artificial intelligence technology, deep learning has demonstrated formidable advantages in the field of mechanical fault diagnosis. Zhao et al. [6] systematically reviewed deep learning research in fluid machinery, clarifying its application directions for fault diagnosis in valves and other fluid machinery, thereby providing a clear theoretical framework for intelligent diagnosis research. Khaleghi et al. [7] focused on the key technology of multi-sensor data fusion, systematically constructing a multi-source information fusion methodology for mechanical fault diagnosis across three integration levels: data layer, feature layer, and decision layer. Notably, Recently, for fault diagnosis under complex operating conditions, Umar et al. [8] proposed a burst-informed acoustic emission framework. This approach employs directional denoising and adaptive frame segmentation to extract multi-domain features, combined with an ensemble classifier to achieve interpretable fault diagnosis for milling machines. Its processing logic for non-stationary signals like acoustic emissions and its multi-domain feature integration strategy provide valuable insights for the systematic fusion of multi-source signals (vibration, pressure) in hydraulic valves. Li et al. [9] utilized wavelet packet transform to extract multi-source features encompassing time, frequency, and time-frequency domains. Their model achieved accurate prediction of internal leakage in check valves under varying pressures, fault severities, and fault categories, fully demonstrating the role of multi-source information in enhancing valve leakage detection. Wang et al. [10] employed a VAF algorithm based on 1D-CNN for bearing fault diagnosis. Compared to traditional “FFT” and “SVM” methods, this approach not only eliminates cumbersome feature engineering steps but also achieves an accuracy rate of 99.33% at SNR=-6 dB, providing an efficient algorithmic foundation for real-time detection of bearing faults in industrial settings. However, most of these methods are designed for specific valve types or scenarios, lacking a universal diagnostic framework applicable across valve types. This limitation restricts their broader adoption in complex and diverse industrial environments.

Regarding deep learning model architectures, the Long Short-Term Memory (LSTM) network proposed by Hochreiter et al. [11] enables effective capture of long-term dependencies in sequential data. This structure provides a technical foundation for achieving high accuracy and robustness in valve leakage detection. Liu et al. [12] systematically reviewed AI applications in rotating machinery fault diagnosis, comparing traditional machine learning with deep learning in terms of fault recognition accuracy, generalization capability, and computational cost, thereby guiding the development of intelligent valve leakage detection systems. Wang et al. [13] proposed a hybrid CNN-LSTM model based on multi-attribute time series data such as system pressure. Applied to fault identification and localization of quick-switching valves in an equal-encoding digital hydraulic system (EDHS), this model achieved an average accuracy of 98.68%. It meets the stringent requirements for fault diagnosis precision in digital hydraulic systems, thereby supporting system fault tolerance. Guo et al. [14] developed a CNN-LSTM deep learning model for fault diagnosis of the hydraulic system in the blanket transfer device of the CFETR. This model achieved a maximum diagnostic accuracy of 98.56% on the test set across four typical fault scenarios, providing technical reference for fault prediction and health management (PHM) of this hydraulic system, as well as for research on intelligent decision-making systems for remote operation of the CFETR. Tong et al. [15] employed multi-scale signal analysis to achieve precise detection of check valve leaks. Compared to single-scale analysis, this approach significantly enhanced the ability to distinguish different leak severity levels, providing technical support for developing graded early warning and maintenance strategies for valve leaks. Li et al. [16] proposed the CFOA-CNN-BiLSTM-Attention hybrid model, which integrates multiple technical advantages to excel in high-precision prediction tasks. Its architectural approach offers valuable insights for high-precision time-series prediction problems like valve leakage. Similarly, the hybrid deep learning framework for milling machines proposed by Siddique et al. [17] effectively addresses core challenges in industrial scenarios—such as noise interference, sparse labeling, and operational variability—by employing baseline drift removal, constructing log-continuous wavelet scale maps, and extracting features via a dual-branch encoder. This further validates the superiority of hybrid architectures in complex mechanical fault diagnosis. However, it is noteworthy that existing research often treats fault classification (qualitative) and leakage rate prediction (quantitative) as two independent tasks, failing to effectively mine and utilize the intrinsic correlations and complementary information between them.

In industrial system fault diagnosis, single-task fault detection has become insufficient for complex scenarios, while the advantages of multi-task collaborative processing are increasingly evident. Chen et al. [18] addressed concurrent faults like pump failures and valve blockages in hydraulic systems by proposing a multi-rate sensor fusion and multi-task learning network. This approach decomposes concurrent fault diagnosis into specific subtasks. Through automatic channel filtering, dual-stream feature extraction, and uncertainty weighting reduction, it achieves simultaneous identification and classification of multiple hydraulic component faults. Its diagnostic performance significantly outperforms traditional methods and other popular multi-output approaches. In other industrial domains like wastewater treatment, Ba-Alawi et al. [19] employed an explainable deep multi-task learning autoencoder network (DMTL-UNet) to simultaneously perform sensor fault diagnosis and reconstruction. Using real-world data from a South Korean wastewater treatment plant, they achieved an F1 score of 99.08% and RMSE reconstruction accuracy of 31.1175 mg/L, while delivering significant energy savings of 37.44%. Moreover, with the advancement of next-generation AI technologies, cutting-edge approaches such as multimodal fusion and Transformer architectures have emerged in the field of industrial intelligent diagnostics. The CNC-VLM model proposed by Wang et al. [20] effectively addresses challenges such as sample imbalance and visual-textual semantic inconsistencies in CNC machine fault detection through RLHF optimization and multimodal learning. It not only achieves high-precision fault recognition but also automatically generates diagnostic descriptions and maintenance recommendations, significantly expanding the practical boundaries of fault diagnosis. The ViT semi-supervised fault diagnosis framework proposed by Siddique et al. [21] proposed a ViT semi-supervised fault diagnosis framework. This framework extracts time-frequency features from sequential signals via continuous wavelet transform and integrates uncertainty quantification with knowledge distillation to address the scarcity of labels in industrial settings. It achieved a 99.68% accuracy rate in milling tool fault diagnosis. Its capability in identifying minor faults and industrial adaptability provides valuable insights for hydraulic valve leakage diagnosis. Although technologies like multi-task learning, multimodal fusion, and Transformer architectures have demonstrated value in hydraulic components, machine tools, and milling cutters, existing research faces clear limitations in the specific context of hydraulic valve leakage diagnosis: On one hand, most methods remain focused on deep mining of single-modal signals like vibration or pressure, lacking systematic mechanisms for fusing multi-source heterogeneous signals; On the other hand, while these cutting-edge techniques show promise, there remains insufficient attention to developing intelligent models that integrate multimodal features with hydraulic domain knowledge while simultaneously adapting to both quantitative leakage prediction and qualitative classification across valve types. Consequently, current research has not kept pace with the latest trends in industrial intelligent diagnostics.

Aiming at the problems of insufficient cross-valve type generalization, lack of complementary synergy of multi-source heterogeneous signal fusion, and separation of quantitative regression and qualitative classification tasks in the existing hydraulic valve leakage diagnosis, this paper innovatively proposes a multi-task learning model based on feature deep fusion (CLAF-MTL), which realizes the high-precision regression of cross-valve-type leakage rate and accurate classification of faults through the complementary fusion of deep automated features and multidomain physical a priori features. type accurate classification for simultaneous prediction.

The main innovations and contributions are as follows:

1. To address the specific requirements for cross-valve-type leakage diagnosis in hydraulic valves, this study innovatively integrates leakage rate regression (quantitative characterization) with fault type classification (qualitative identification) into a unified modeling framework. The core architecture comprises “CNN-LSTM-Additive Attention + Multi-domain Feature Fully Connected Network (FCN)”. Compared to existing multi-task learning approaches for hydraulic systems, this work overcomes two critical limitations: First, existing multi-task models predominantly focus on concurrent fault classification across multiple components or sensor signal reconstruction. They lack a “quantitative + qualitative” dual-task coordination mechanism tailored for hydraulic valve leakage scenarios, failing to meet the integrated industrial demand for “fault identification + leakage quantification” Second, traditional multi-task approaches often employ fixed weighting strategies, leading to “dominant optimization of one task while the other degrades” This paper dynamically optimizes dual-task weights via the MGDA-UB algorithm, calculating gradient direction consistency only for shared layer parameters. This reduces computational complexity while achieving collaborative gradient matching and complementary parameter updates across tasks. Furthermore, it leverages CNNs to precisely capture local transient signal features, bidirectional LSTMs to deeply mine long-range temporal dependencies, and an Additive Attention mechanism to adaptively focus on critical leakage period information. Combined with multi-domain feature FCN, this forms an efficient collaborative inference mechanism of “data-driven feature mining + domain knowledge-guided constraints” This approach overcomes the application limitations of existing multi-task models in hydraulic valve leakage diagnosis by enhancing scenario adaptability and architectural design.
2. Breaking through the limitations of traditional single-modality or single-dimensional feature extraction, this approach systematically integrates “deep auto-features” and “multi-domain manual features” derived from dual-modality vibration and pressure signals: Deep auto-features autonomously capture complex nonlinear leakage evolution patterns through deep learning networks—patterns difficult for humans to characterize. Multi-domain manual features extract domain-specific a priori characteristics with clear physical significance through time domain, frequency domain, and wavelet packet domain (total entropy + sub-band energy ratio) analysis. The complementary integration of these two feature types overcomes the expressive limitations of single-modality features while addressing the interpretability shortcomings of pure deep learning models. This enables the model to accurately identify complex leakage patterns while anchoring fault essence to physical features, significantly enhancing robustness and generalization capabilities.
3. Breaking through the compatibility bottleneck of traditional diagnostic methods being “single-valve-type specific” this innovation comprehensively covers three typical industrial valve types: check valves, globe valves, and solenoid ball valves. It specifically addresses the challenge of feature adaptation caused by significant structural differences and complex failure mechanisms across various valve types. Through synchronous multi-sensor data acquisition and multi-dimensional data augmentation strategies, it effectively overcomes technical challenges in cross-valve-type diagnostics—such as imbalanced fault samples and heterogeneous feature distributions—delivering a comprehensive technical solution for valve condition monitoring in complex industrial environments.

## 2. Methodology

### 2.1. CLAF Sequence Prediction Architecture

The CLAF architecture centers on dual-path feature extraction and complementary fusion to specifically address the critical challenges posed by significant structural variations among valve types and heterogeneous fault characteristics. Through synergistic collaboration, its modules provide unique support for cross-valve generalization. Simplified, the first path comprises the CNN-LSTM-Additive Attention module, which focuses on data-driven discovery of cross-valve common features: CNN captures local transient patterns of various valve leaks through multi-scale convolutions, while bidirectional LSTM uncovers long-range temporal dependencies in leakage processes, adapting to the dynamic evolution of leaks across valve types. The Additive Attention mechanism adaptively focuses on critical time intervals, reducing interference from valve type variations. This tripartite collaboration liberates the model from the constraints of valve-specific feature sets, enabling it to learn universal leakage characteristics across valve types. The second branch comprises a Multi-domain Feature Fully Connected Network (FCN), emphasizing domain-knowledge-guided specificity constraints. By extracting physical features from the time domain, frequency domain, and wavelet packet domain, it anchors the fundamental failure characteristics of different valve types. The deep fusion of these two feature types ensures both cross-valve-type diagnostic generality and single-valve-type specificity, laying a solid foundation for the model’s generalization performance. The core logic of this dual-path collaboration clearly outlines the overall framework of the CLAF architecture. The following sections will elaborate on the model’s specific structural design and implementation details.

The research employs PyTorch (Version 1.13.0), a deep learning framework developed by Facebook’s AI Research team. The computer configuration consists of an Intel Core i5-9300H CPU (Intel Corporation, Santa Clara, CA, USA) with 8 GB of RAM and an NVIDIA GeForce GTX 1660 Ti (NVIDIA Corporation, Santa Clara, CA, USA) graphics card with 6 GB of memory. A CNN-LSTM-Additive Attention model was constructed using Python (Version 3.9) within the PyTorch environment.

The CLAF model was established by incorporating the CNN-LSTM-Additive Attention model and a fully connected neural network, following the methodology described in Reference [16]. The CNN module comprises three convolutional blocks, each consisting of a one-dimensional convolutional layer, a batch normalization layer, a ReLU activation function, and a max pooling layer. The input layer utilizes dual-channel inputs of pressure and vibration signals. The convolution kernel size progressively decreases from 7→5→3, enabling multi-scale feature capture of macro-scale fluctuations, meso-scale variations, and micro-scale transients within the signals, thereby accommodating leakage features of varying durations. Channel expansion follows an incremental strategy of 32→64→128 channels, enabling the model to learn more complex feature combinations as the network deepens, aligning with the hierarchical nature of feature abstraction. The convolutional layer employs the ReLU activation function to mitigate the vanishing gradient problem. To prevent overfitting, Dropout is adopted as the core regularization technique across critical modules. A uniform dropout rate of 0.3 is applied throughout the network, synchronously implementing random neuron dropout at four key positions: after CNN convolutional blocks, between LSTM layers, and within the feature fusion layer of the feature processing network.

This study employs Long Short-Term Memory (LSTM) networks as the core temporal modeling unit. Through its gating mechanisms (forget gate, input gate, and output gate), LSTM dynamically regulates cell states to effectively capture long-range temporal dependencies between pressure and vibration signals during leakage events—such as the delayed correlation between gradual pressure decline and vibration impact signals. To further enhance the model’s perception of temporal context, a bidirectional LSTM architecture is employed. This simultaneously integrates forward and backward sequence information, thereby more comprehensively characterizing the dynamic evolution patterns of vibration and pressure signals during leakage.

**Figure 1:**
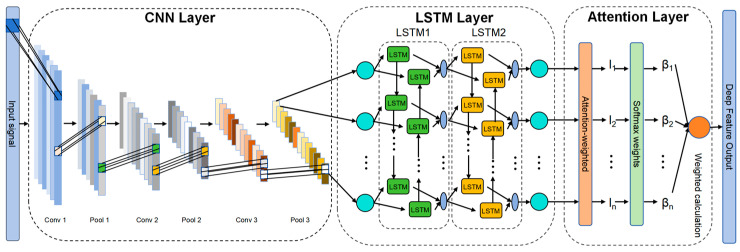
CNN-LSTM-Additive Attention Model. The color coding in the figure represents different components of the CNN-LSTM-Additive Attention model, including input signals, convolutional feature maps, LSTM gate units, attention weights, and the final fused feature vector.

Attention mechanisms were introduced to optimize the model’s ability to focus on key features. Although the hidden states at each time step processed by LSTM contain all temporal information, their contributions to the final leakage diagnosis and quantification prediction vary. This model incorporates a Soft Attention Mechanism, which calculates attention weights for each time step using a learnable parameter matrix to generate weighted context vectors. This mechanism enables the model to autonomously focus on the critical time periods most relevant to leaks while suppressing interference from non-essential temporal information. Consequently, it significantly enhances the model’s feature utilization efficiency and diagnostic robustness. The model architecture, as shown in Figure 1, employs a cascaded CNN-LSTM-Additive Attention structure to achieve a complete workflow: from local feature extraction of raw signals, through modeling long-range temporal dependencies, to focusing on key information.

**Figure 2:**
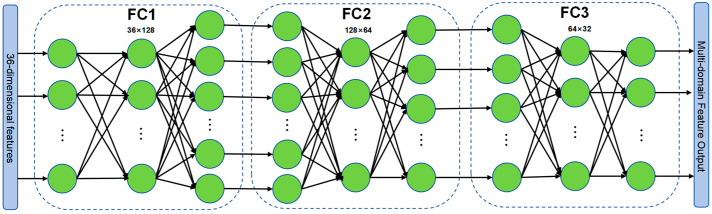
Multi-domain Feature Extraction Model. The color coding in the figure represents different components of the multi-domain feature extraction model, including the input/output feature vectors, neurons in fully connected layers, weighted connections.

Another branch is the multi-domain feature extraction model, as shown in Figure 2. It extracts temporal, frequency, and wavelet domain features from vibration and pressure signals, infuses domain-specific prior knowledge, and then performs feature dimensionality reduction through a fully connected network. This process outputs concatenated features that can be fused with deep network features. This approach enhances model interpretability through prior knowledge, complements the abstract temporal features extracted by CNN-LSTM-Additive Attention to improve generalization performance, and leverages the flexible adaptability of fully connected structures to balance expressive power and training efficiency.

This paper innovatively employs a complementary fusion mechanism between multi-domain features and deep automatic features. This approach leverages the domain-specific prior knowledge embedded in multi-domain features to provide the model with physically meaningful fault anchors, enhancing interpretability and robustness for conventional leakage patterns while preventing deep networks from deviating from core diagnostic logic due to data noise. Simultaneously, it leverages deep auto-features extracted via CNN-LSTM-Additive Attention to capture complex nonlinear patterns difficult for humans to design. This approach adapts to diverse leakage scenarios and unknown fault evolution patterns, enhancing model interpretability while improving generalization performance under complex operating conditions.

### 2.2. Multi-Task Learning Framework

**Figure 3:**
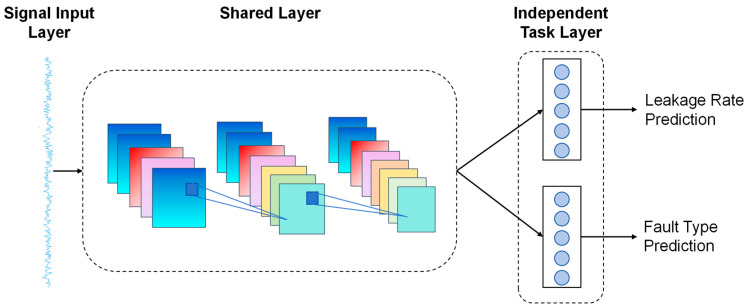
Multitask Learning Framework. The color coding in the figure represents different components of the multi-task learning framework, including input signals, multi-scale feature maps, task-specific neurons.

The proposed multi-task learning framework centers on “feature sharing-task collaboration-joint optimization” to achieve end-to-end joint modeling for valve leakage prediction and fault category identification. As illustrated in Figure 3, this framework adopts a dual-input-single-shared-layer-dual-output-head architecture. The input layer simultaneously receives raw signals from vibration and pressure channels alongside multi-domain features, generating complementary features through two parallel feature extraction chains: The first is a CNN-LSTM-Additive Attention module. Multi-scale convolutions capture local transient patterns in signals, bidirectional LSTMs uncover long-range temporal correlations, and attention mechanisms focus on critical time intervals, yielding deep automatic features embodying abstract evolutionary patterns. The second is a fully connected network that performs dimensionality reduction and nonlinear transformations on time-domain, frequency-domain, and wavelet-domain features, outputting domain-specific prior features. These two feature types fuse at the shared layer to form a unified representation that combines data-driven patterns with domain knowledge constraints. Dual output heads perform differentiated predictions based on the shared features: The regression head outputs continuous leakage values based on continuous evolutionary features, while the classification head outputs probability distributions of fault categories using category-specific features. Parameter coordination is achieved through a weighted joint loss. This approach enables the classification task to provide category prior constraints for regression, while the regression task enhances the classification task’s ability to distinguish degrees of severity. Ultimately, this improves single-task accuracy while boosting model generalization and efficiency through feature reuse.

To address the limitations of single-task modeling—including weak generalization capabilities, isolated task information, and the tendency for fixed weights in multi-task settings to lead to “dominant optimization of one task while the other’s performance degrades”—this framework adopts a core optimization objective: synergistically enhancing valve leakage prediction accuracy and fault identification accuracy while balancing the optimization priorities of both tasks. By leveraging feature sharing to uncover task correlations and dynamically adjusting weights during training, it achieves mutually beneficial constraints and efficient collaboration between the two task types.

Let $\omega_{i}$ denote the weight of the *i*-th task, and *N* denote the number of tasks. Then the optimization objective for the total multi-task loss can be expressed as:

(1) $$L_{MTL}=\sum_{i=1}^{N}\omega_{i}L_{i}$$

Optimizing the above equation using a stochastic gradient descent-type algorithm, the update formula for the shared layer parameters is:

(2) $$W_{sh}=W_{sh}-\gamma \sum_{i=1}^{N}\omega_{i}\frac{\partial L_{i}}{\partial W_{sh}}$$

In the equation, $W_{sh}$ denotes the shared layer network parameters; γ represents the learning rate. As shown above, when the gradient value for a particular task is large, the update of shared layer parameters becomes dominated by that task, thereby preventing other tasks from being optimized effectively.

For the dual tasks of valve leakage regression (*reg*) and fault classification (*cls*), the multi-task loss can be further refined into a joint function of shared parameters and task-specific parameters:

(3) $$L_{m}\left(\theta_{sh},\theta_{r},\theta_{c}\right)=\omega_{r}L_{r}\left(\theta_{sh},\theta_{r}\right)+\omega_{c}L_{c}\left(\theta_{sh},\theta_{c}\right)$$

In the formula, $\omega_{r}$ and $\omega_{c}$ represent the base weight configurations for the regression head and classification head, respectively. $\theta_{sh}$ denotes the shared layer parameters, $\theta_{r}$ denotes the regression head parameters, and $\theta_{c}$ denotes the classification head parameters.

The training optimization of the entire multi-task learning network can be defined as:

(4) $$\theta =\arg\min_{\theta_{sh},\theta_{r},\theta_{c}}L_{m}\left(\theta_{sh},\theta_{r},\theta_{c}\right)$$

To address task imbalance caused by static weights, the MGDA-UB algorithm dynamically optimizes dual-task weights. Its core principle involves solving for optimal weights that align the gradient directions of shared layer parameters for regression and classification tasks, expressed as:

(5) $$\omega^{*}=\arg\min_{\omega_{r},\omega_{c}}\left\|\omega_{r}\cdot \nabla_{W_{sh}}L_{r}+\omega_{c}\cdot \nabla_{W_{sh}}L_{c}\right\|2$$

In the equation, $\omega^{*}$ denotes the optimized dual-task weight combination obtained through optimization. $\nabla_{W_{sh}}L_{r}$ represents the gradient of the regression task loss $L_{r}$ with respect to the shared layer parameters $\nabla_{W_{sh}}$. $\nabla_{W_{sh}}L_{c}$ denotes the gradient of the classification task loss $L_{c}$ with respect to the shared layer parameters $\nabla_{W_{sh}}$.

Compared to traditional multi-task optimization methods with fixed weights, MGDA-UB offers advantages better suited to practical needs in valve diagnostics. First, by adaptively adjusting weight distribution based on gradient information, it perfectly accommodates the dynamic difficulty shifts between regression and classification tasks inherent in valve diagnostics. Second, the algorithm avoids redundant calculations by computing gradients only for shared layer parameters, rather than performing full-chain gradient computations for each task. This significantly reduces computational redundancy when handling high-dimensional inputs—such as long dual-channel signals (vibration and pressure) combined with 36-dimensional multi-domain features—effectively lowering computational complexity and overall training overhead. Third, dynamic weight adjustment enables precise task balancing across training phases: During early training, it balances the large gradients of the classification task with the gentle gradients of the regression task, preventing dominance by a single task. In later training, it prioritizes fine-tuning the regression task, accelerating convergence of leakage prediction errors and ultimately achieving synergistic performance improvements for both tasks.

### 2.3. Wavelet Decomposition

Wavelet packet decomposition is widely used in fault signal analysis due to its ability to provide finer frequency resolution. It involves decomposing an input signal into multiple scales using a set of orthogonal wavelet bases, with the decomposition formula expressed as:

(6) $$\left\{\begin{array}{l} d_{j+1}^{2k}=\sum_{m}h\left(m\right)d_{j}^{k}\left(2n-m\right)\\ d_{j+1}^{2k+1}=\sum_{m}g\left(m\right)d_{j}^{k}\left(2n-m\right) \end{array}\right.$$

Here, $d_{j}^{k}\left(n\right)$ denotes the wavelet packet coefficient at node *k* in layer *j*, *n* represents the offset in the filtering operation, $h\left(m\right)$ and $g\left(m\right)$ are the lowpass and highpass filter coefficients respectively (satisfying orthogonality), *j* is the decomposition level, and *k* is the node index.

In selecting the wavelet basis and decomposition levels, this study comprehensively considered the characteristics of hydraulic valve leakage signals and the practicality of the method. Ultimately, the db4 wavelet basis and a 3-level decomposition hierarchy were adopted. The specific selection rationale and a brief comparison with commonly used wavelet bases are as follows:

Among commonly used wavelet bases, the db series (db1–db10), sym series (sym4–sym8), and coif series (coif1–coif5) are all widely applied in mechanical fault diagnosis, each with distinct strengths: db1 (Haar wavelet) is computationally simple but lacks sufficient smoothness, limiting its ability to capture transient impact features in leakage signals; Higher-order db wavelets like db8 offer superior smoothness and time-domain localization, but their computational complexity increases significantly, and they may lose subtle leakage-related features due to excessive smoothing. Sym and coif wavelets exhibit better symmetry and lower signal reconstruction errors, but their frequency-domain resolution for non-stationary vibration signals is slightly inferior to the db series. In contrast, the db4 wavelet basis combines moderate smoothness with good time-frequency localization properties. It can accurately capture transient impact signals caused by leakage while effectively distinguishing differences in frequency-domain energy distribution corresponding to varying leakage levels. Additionally, its computational complexity is moderate, making it suitable for the dual-modal signal processing requirements of this study. This aligns with the established application verification of the db4 wavelet basis in mechanical fault diagnosis [9].

**Figure 4:**
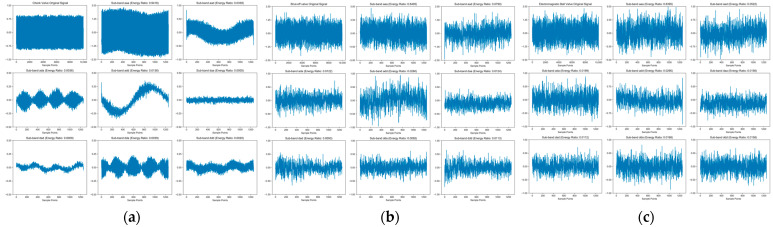
Orthogonal sub-frequency bands after wavelet decomposition of various valves. (**a**) Check valve. (**b**) Shut-off valve. (**c**) Electromagnetic Ball Valve.

Regarding decomposition levels, a three-level decomposition precisely covers the critical 0–1 kHz frequency band, yielding eight orthogonal sub-bands (corresponding to the Nyquist frequencies of the downsampled signal). This approach avoids insufficient feature information from lower-level decomposition while preventing redundant sub-bands from higher-level decomposition, thereby enhancing feature extraction efficiency. The three orthogonal frequency bands with different thresholds are shown in Figure 4. Similar studies have demonstrated the effectiveness of the “wavelet packet energy ratio + total entropy” feature for quantifying leakage rates [22]. This paper adopts the 9-dimensional feature vector extracted from the three-level decomposition results of the db4 wavelet basis, including: wavelet packet total entropy and the energy ratios of the eight sub-bands (aaa to ddd). The total wavelet packet entropy reflects the complexity of the signal’s energy distribution, while the sub-band energy ratios characterize the energy proportion in each frequency band. The calculation formulas are as follows:

(7) $$H=-\sum_{i=1}^{8}p_{i}\log_{2}p_{i}$$

(8) $$p_{i}=\frac{E_{i}}{\sum_{j=1}^{8}E_{j}}$$

The energy of the *i*-th subband is the energy ratio of the *i*-th subband, where *H* is the total entropy.

### 2.4. Data Downsampling and Prediction Methods

First, the signals underwent downsampling processing using the resample function based on an FIR anti-aliasing filter. This not only uniformly reduced the vibration and pressure signals from their original sampling rate to 2 kHz but also ensured signal integrity without distortion within the 0–1 kHz analysis band. This coverage encompasses the primary energy concentration region of vibrations caused by valve leakage.

**Figure 5:**
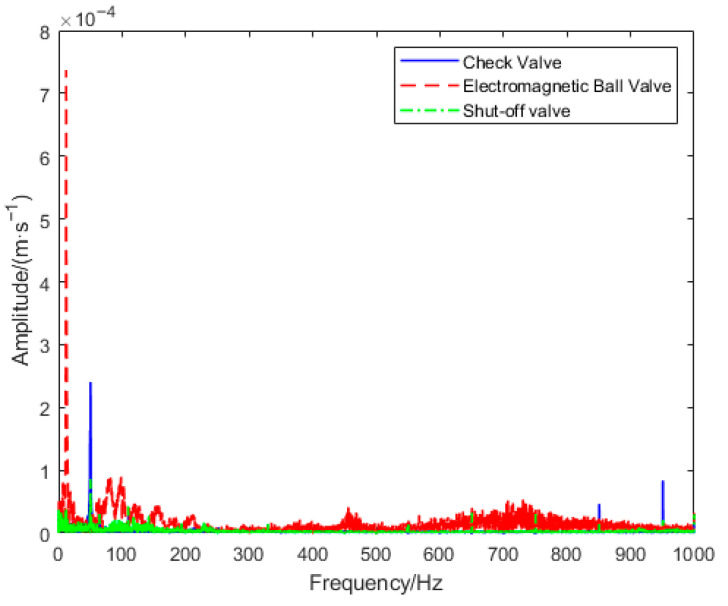
Vibration Signal Spectrum Comparison Chart for Various Valves at Similar Leakage Rates.

Similar studies indicate that after anti-aliasing with a cutoff frequency of 1 kHz, there is virtually no energy above this threshold [23]. Therefore, 1 kHz can be adopted as the cutoff frequency for anti-aliasing filtering. Figure 5 displays the vibration signal spectra of three different valves at similar leakage rates, revealing that the spectra are concentrated within the 0–1 kHz range of the spectrum diagram. This paper employs a Fourier transform-based frequency domain resampling method for signal downsampling. This approach converts the original signal to the frequency domain via discrete Fourier transform, retains key fault feature frequencies within the 0–1 kHz range using frequency domain truncation principles, and reconstructs the downsampled signal through inverse transform.

**Figure 6:**
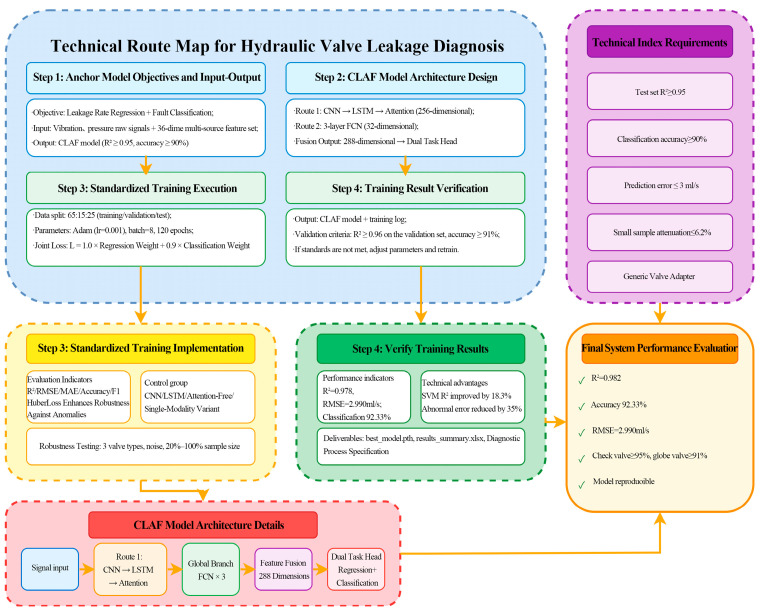
CALF-MTL Model Technology Roadmap.

The preprocessed dual-channel vibration-pressure raw signal sequence undergoes multi-scale local feature extraction via the aforementioned three convolutional blocks. This sequence is then fed into a two-layer bidirectional LSTM to learn long-range temporal dependencies in the leakage process. An attention mechanism subsequently generates context vectors focused on critical time intervals, enabling precise capture of core information. Concurrently, a 36-dimensional multi-domain feature vector undergoes nonlinear transformation and dimensionality reduction through a dedicated fully connected network (FCN). Finally, the attention-output context vector is concatenated with the transformed multi-domain features. The resulting fused features are input into two task-specific output heads: the regression head outputs continuous leakage rate predictions, while the classification head outputs probability distributions for valve faults. This end-to-end process from raw signals to dual-task predictions fully leverages multi-source information fusion and deep learning advantages. The workflow is illustrated in Figure 6:

## 3. Experiment

The experimental setup consists of two components: a fault simulation test bench and a self-built fault signal acquisition system. Valve internal leakage faults are simulated using the RCYCS-G hydraulic system fault diagnosis bench. This bench comprises a hydraulic pump, pressure gauge, solenoid ball valve, check valve, globe valve, measuring cup, hydraulic lifting platform, and accumulator. The accumulator regulates the pressure upstream of the valves, supplying energy to the three valve types used in the experiment. Pre-processing was performed on the check valve spool, while pre-treatment was applied to the globe valve and solenoid ball valve. The specific valve types, fault categories, and their corresponding designations are detailed in Table 1.

Measure the vibration and pressure signals of various valves under different conditions as shown in Table 2. Prior to testing, charge the accumulator using a hydraulic pump and adjust its pressure to 6.0 MPa. Under this operating pressure, collect pressure, vibration, and temperature signals for each fault condition. Calculate the internal leakage rate of different valves under fault conditions using the measuring cup method. Each fault condition for every valve type was tested 30 times, yielding a total of 330 sets of experimental data.

**Figure 7:**
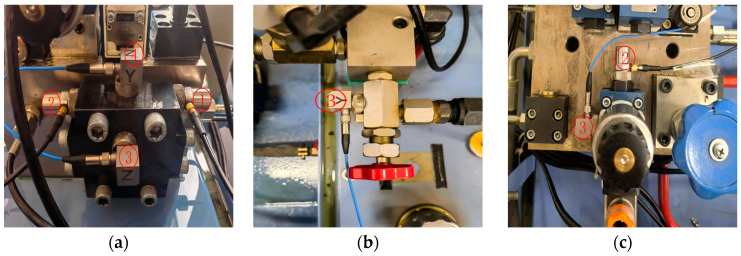
Sensor placement in the experiment. (**a**) Check valve. (**b**) Shut-off valve. (**c**) Electromagnetic Ball Valve. The red-circled numbers represent the sensor IDs used at the measurement points.

This fault acquisition system comprises an NI Pxle-1062Q data acquisition chassis (National Instruments Corporation, Austin, TX, USA), an NI Pxle-8840 controller (National Instruments Corporation, Austin, TX, USA), an NI Pxle-4462 data acquisition module (National Instruments Corporation, Austin, TX, USA), vibration sensors, and LABVIEW (Version 2018) software. All four sensors utilize IEPE piezoelectric accelerometers (Jiangsu Donghua Testing Technology Co., Ltd., Jingjiang, China), with a sampling frequency set to 20 kHz for all sensors. Detailed sensor specifications are listed in Table 2:

The vibration sensor arrangements for each measured valve are shown in Figure 7. For the check valve, sensors 1, 2, 3, and 4 are used as depicted in Figure 7a. For the globe valve, sensor 3 is used as shown in Figure 7b. For the solenoid ball valve, sensors 2 and 3 are used as illustrated in Figure 7c.

## 4. Test Signal Analysis and Processing

### 4.1. Analysis and Processing

To meet the CNN-LSTM-Additive Attention model’s requirement for consistent input dimensions, a random sliding window continuously captures 10,000 data points, preventing potential loss of valid signals that might occur if only the signal’s beginning were sampled. Signals shorter than the required length are symmetrically zero-padded to meet the length requirement while preserving signal morphology.

Robust normalization is employed to eliminate the effects of sensor dimensions and baseline drift, suppress interference from individual outliers, and enhance the stability and convergence speed of model training. This method utilizes the median and interquartile range as statistical measures:

(9) $$z=\frac{x\;-\;M}{IQR+\varepsilon }$$

where *z* is the normalized signal, *x* is the signal, and *M* is the median.

The median represents the central tendency of data, while the IQR measures statistical dispersion. Both are insensitive to outliers, thereby ensuring the robustness of normalization results.

### 4.2. Multi-Domain Feature Extraction

To enhance model interpretability and effectively complement depth, this study extracts features from preprocessed signals. Sixteen-dimensional time-frequency domain features (9-dimensional time domain features and 7-dimensional frequency domain features) are directly extracted from the vibration signals. Simultaneously, the original signals undergo wavelet packet decomposition to extract a 9-dimensional feature vector, resulting in a total of 25-dimensional vibration features.

**Figure 8:**
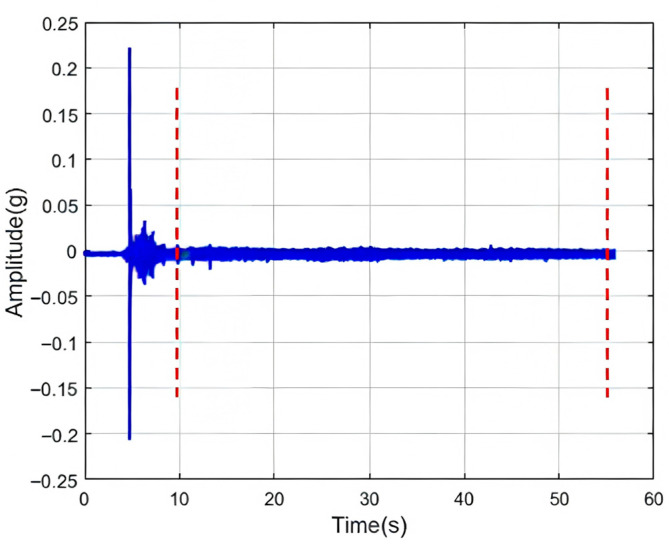
Time-domain plot of the original signal. The red dashed line indicates the valid segment of the intercepted signal.

To avoid the influence of transient processes during valve opening and the final stage of leakage, calculations were performed using signals from the intermediate 80% steady-state leakage phase. The original signal is shown in Figure 8. This study extracted 11 features based on both the steady-state characteristics and the rate of change in dynamic characteristics of the pressure signal.

**Figure 9:**
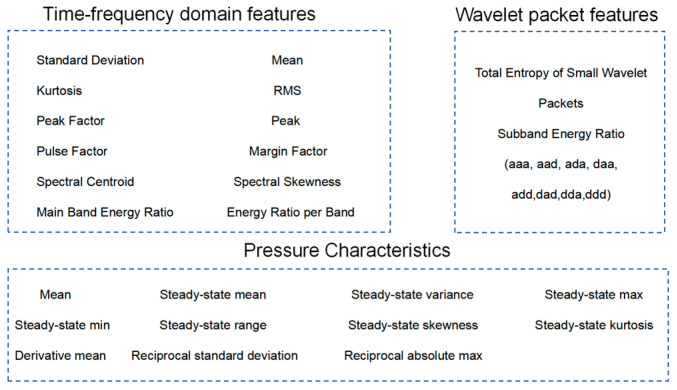
Multi-domain Feature Extraction Summary for Pressure and Vibration Signals.

The multi-domain feature vector for each sample is constructed by concatenating 25-dimensional vibration features and 11-dimensional pressure features. Following the principle of multi-source feature complementarity, this forms a 36-dimensional high-dimensional feature vector [24], as shown in Figure 9. This feature set comprehensively describes the valve’s operational state from multiple physical perspectives. Together with the raw signal sequences from both channels, it serves as input to the model, achieving effective integration of feature engineering and deep learning.

### 4.3. Data Augmentation Strategy

To address the scarcity of fault samples and the imbalance in sample sizes across different valve fault types encountered in this study, targeted data augmentation techniques were introduced during the training phase to suppress model overfitting and enhance its generalization capability. This study comprehensively employs three data augmentation strategies: random time shifting, random amplitude scaling, and Gaussian noise addition. These aim to significantly improve the model’s generalization and robustness under the uncertainties of real industrial environments. This approach draws inspiration from techniques addressing strong noise, high-dimensionality, and highly nonlinear features [25]. Specifically: Random time shifting (maximum offset ±15%) simulates uncertainty in leakage onset timing caused by solenoid valve response and mechanical delays by perturbing the signal’s initial phase. This forces the model to focus on learning relative waveform characteristics and dynamic patterns of the leakage process rather than absolute temporal localization; Random amplitude scaling (scaling factor α ∈ [0.8, 1.2]) simulates signal amplitude fluctuations caused by varying sensor sensitivities and installation conditions. This effectively prevents model overfitting to superficial absolute amplitude features, guiding it to learn more intrinsic normalized shapes and frequency-domain patterns; Adding Gaussian noise (standard deviation σ = 0.01) yields a signal-to-noise ratio of approximately 37.4 dB. This precisely introduces random perturbations simulating inherent electromagnetic interference and measurement noise in real industrial environments, enhancing the model’s robustness against background noise and preventing its reliance on overly idealized clean data.

The synergistic application of these three technologies systematically expands the distribution range of training data across three critical dimensions—temporal phase, amplitude scale, and signal purity—while integrating domain knowledge into the training process. This collaborative strategy collectively ensures that model decisions are grounded in robust features intrinsic to the physical nature of leakage, rather than irrelevant measurement variables. Consequently, it significantly enhances the model’s predictive accuracy and reliability in unknown, noise-rich real-world industrial scenarios.

To ensure the objectivity of model training and the unbiased nature of evaluation results, this study strictly adheres to the principle of “data partitioning before data augmentation” The 519 original samples were stratified by valve type and fixedly divided into training (311 samples), validation (78 samples), and test (130 samples) sets at a ratio of 60:15:25. Subsequently, data augmentation was applied to each dataset, ultimately expanding the total sample size to 5043. After augmentation, the training, validation, and test sets comprised approximately 3026, 757, and 1260 samples, respectively. All augmented data generated from the original samples were strictly confined to their corresponding initial datasets, avoiding cross-distribution between datasets. This effectively prevented evaluation bias caused by data leakage, ensuring the fairness and reliability of model performance assessment.

### 4.4. Visual Verification and Analysis of Multimode Characteristics

To visually validate the discriminative effectiveness of sensor-derived features, domain-specific manually designed features, and multi-modal deep fusion features, this study employs two classical dimensionality reduction methods—Principal Component Analysis (PCA) and t-distributed Stochastic Neighborhood Embedding (t-SNE)—to visualize the high-dimensional spatial distributions of automatically extracted features, multi-domain manually designed features, and multi-modal fusion features. Feature performance is evaluated across two core dimensions: valve classification accuracy and clustering compactness of operating condition parameters.

**Figure 10:**
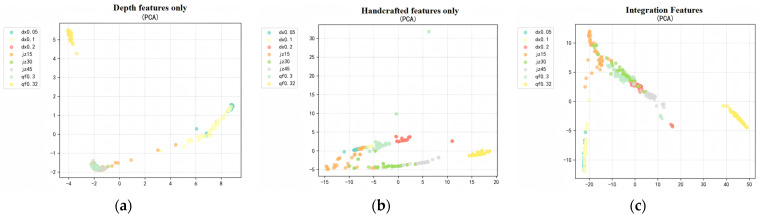
PCA Dimension Reduction Visualization Results. (**a**) Depth features only. (**b**) Handcrafted features only. (**c**) Integration Features.

The PCA linear dimensionality reduction results shown in Figure 10 reveal significant differences in the global distribution of the three feature categories. Only the automatically extracted feature samples exhibit substantial category overlap, with blurred boundaries between different valve types and between operating condition parameters within the same valve type. This indicates limited global characterization capability regarding valve type differences and variations in operating condition parameters. Only the manually selected multi-domain features exhibit relatively dispersed sample distributions. While they can preliminarily distinguish the three major valve types—check valves, globe valves, and solenoid ball valves—sample clusters for different operating parameters within the same valve type still overlap. This reflects the insufficient global discriminative power of manual features for subtle variations in operating parameters. In contrast, multi-modal fusion features exhibit a distinct “valve-type macro-cluster + operating-condition parameter sub-cluster” structure. Check valves, globe valves, and solenoid ball valves each form independent major clusters. Within each major cluster, samples with different faults and operating conditions further aggregate into gradient subclusters. This demonstrates that the fused features effectively integrate both structural differences among valve types and quantitative variations in operating conditions while preserving global variance.

**Figure 11:**
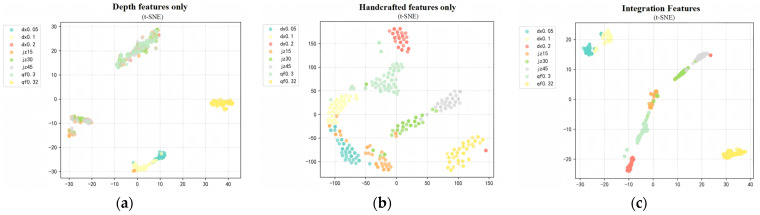
t-SNE Dimension Reduction Visualization Results. (**a**) Depth features only. (**b**) Handcrafted features only. (**c**) Integration Features.

The t-SNE nonlinear dimensionality reduction results shown in Figure 11 further validate the superiority of multi-modal fusion features. The local clustering effect of automatically extracted features alone is mediocre, with partial overlap observed among samples of different valve types, making it difficult to precisely distinguish valve boundaries. This is related to the susceptibility of single automatic features to signal noise and modal aliasing interference. Manually selected multi-domain features enabled each valve type to form independent clusters, but samples within clusters exhibited high dispersion. Boundaries between clusters representing different operating parameters within the same valve type remained blurred. This indicates that while manual features possess physical interpretability, their ability to capture nonlinear signal characteristics is limited. In contrast, samples featuring multi-modal fusion characteristics exhibit a compact, stratified cluster structure with clear separation. Samples representing varying degrees of eccentricity faults in the check valve series cluster sequentially in the upper-left region. Samples of different opening degrees in the globe valve series form gradient subclusters in the central area. While samples of varying return-to-position obstruction levels in the solenoid ball valve series formed an independent subcluster in the lower right. Samples were highly concentrated within clusters with no overlap between them, fully demonstrating the fusion features’ global discrimination capability for valve type differences and their local precision in capturing operating parameter variations.

In summary, the dual validation results from PCA and t-SNE demonstrate that multi-modal fusion features significantly outperform both single auto-extracted features and multi-domain manual features in valve classification accuracy and operational parameter clustering compactness. This effectively demonstrates the complementary synergistic effects of the two feature types: automatically extracted features excel at capturing deep dynamic patterns in signals that are difficult for humans to perceive, while multi-domain manually selected features embody domain-specific prior knowledge about valve structure and fault evolution. After feature-level fusion, the combined approach not only accurately distinguishes between check valves, globe valves, and electromagnetic ball valves, while also detailing the quantitative differences in faults and operating parameters within the same valve category. This provides highly discriminative feature input support for subsequent multi-valve-type, multi-condition leakage rate prediction and fault type identification models.

## 5. Leakage Rate and Valve Type Prediction

### 5.1. Analysis of Leakage Rate and Accuracy in Predicting Valve Failure Types

To comprehensively evaluate the overall performance of multi-task learning models in regression and classification tasks, this study quantitatively analyzes model accuracy using evaluation metrics specific to regression and classification tasks, respectively. First, the multi-task label definitions in this study are clarified, encompassing two core task labels:—Regression task labels: Actual leakage rates (mL/s) of valves under various operating conditions, used to quantitatively characterize leakage severity.—Classification task labels: Specific failure types corresponding to valves, used to qualitatively distinguish failure categories. The overall performance on the test set and the performance broken down by valve failure type are shown in Table 3.

Through an in-depth analysis of Table 3, the following conclusions can be drawn: The model achieved a prediction accuracy of RMSE = 2.990 mL/s and MAE = 2.037 mL/s on the test set. Most importantly, its R^2^ value reached 0.978. This indicates an exceptionally high degree of goodness-of-fit between the predicted values and the actual values, fully validating the overall accuracy and reliability of the model.

**Figure 12:**
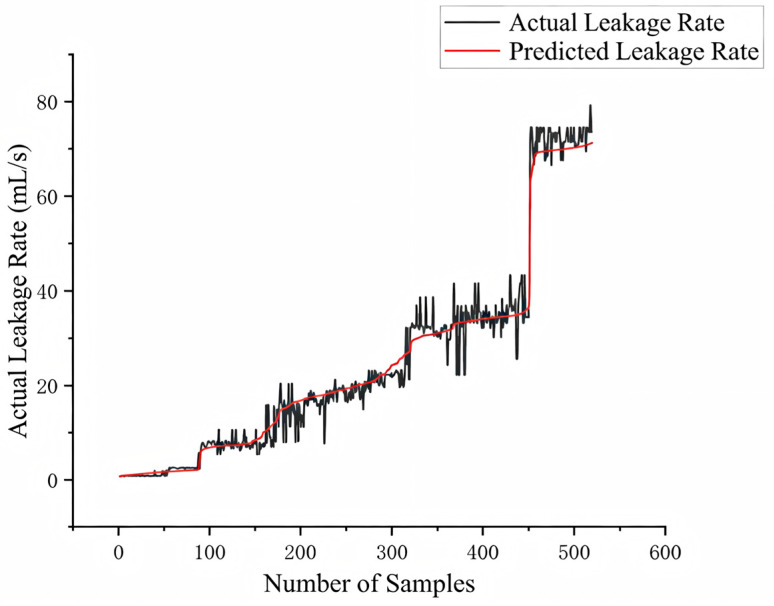
Leakage Rate Prediction Results.

The electromagnetic ball valve demonstrated the most accurate prediction performance: its RMSE (1.622 mL/s) and MAE (0.942 mL/s) were both the lowest, with an R^2^ value approaching 0.99. This indicates that for faults such as the electromagnetic ball valve spool reset obstruction, the model achieves near-perfect prediction accuracy. Prediction errors for check valves and globe valves were relatively higher, with R^2^ values of 0.966 and 0.921, respectively. This may stem from the more complex hydrodynamic phenomena caused by eccentric faults in check valves and opening variations in globe valves, resulting in stronger nonlinear leakage processes that increase prediction difficulty. Prediction results are shown in Figure 12.

**Figure 13:**
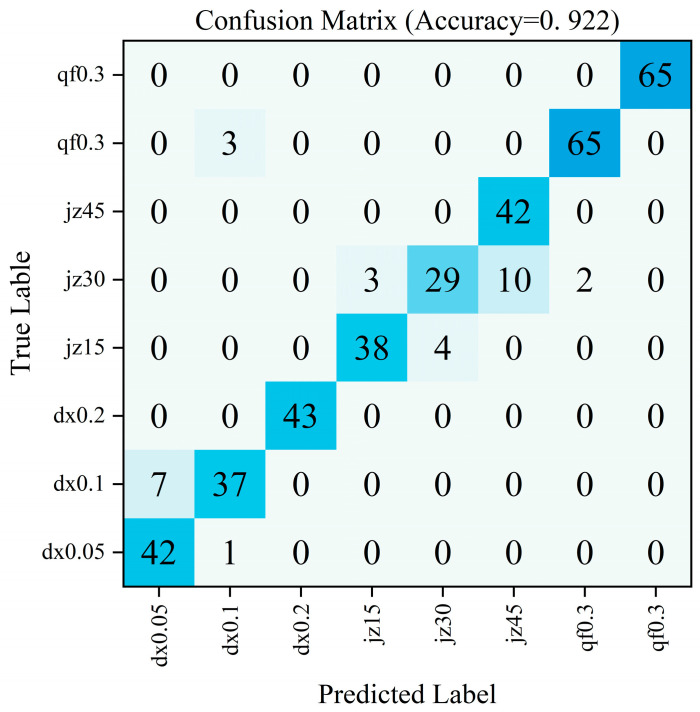
Fault Type Prediction Confusion Matrix.

The model demonstrated strong overall classification performance in diagnosing fault types, achieving an overall accuracy of 92.33% in valve fault classification. The F1-Score for each category exceeded 0.820, indicating balanced high classification performance across all three fault categories. The confusion matrix is shown in Figure 13.

Electromagnetic ball valves demonstrated the most outstanding classification performance (96.5% accuracy, F1-Score 0.975). Their confusion matrix revealed extremely few misclassified samples, indicating that their vibration and pressure signal features differ significantly from the other two valve types, making them easily distinguishable. However, the prediction accuracy for check valves and globe valves is relatively lower (check valve accuracy: 89.8%, globe valve accuracy: 89.2%). Analysis of the confusion matrix reveals a small number of samples misclassified into other categories. This may be attributed to certain operational conditions where their vibration signal patterns exhibit similarities, thereby increasing classification difficulty.

### 5.2. Experimental Study on Melting

To validate the necessity and synergistic effects of each core component in the CLAF-MTL model, this study designed ablation experiments based on the principle of controlling variables: Fixed dataset partitioning, training hyperparameters (Adamw optimizer, initial learning rate 0.001, batch size 8, training epochs 120), and evaluation metrics, we constructed six model variants by sequentially removing or replacing key modules. By comparing the changes in dual-task performance, we precisely identified the functional value of each component. Experimental results are shown in Table 4.

The experimental results demonstrate that the omission or replacement of any core component leads to varying degrees of model performance degradation. Moreover, the roles of key components exhibit a pronounced hierarchical structure:

From the basic LSTM model to the CNN-LSTM model, and then to the complete CNN-LSTM-Additive Attention model, the RMSE for leakage rate prediction decreased by 13.2% and 53.1% respectively, while the R^2^ value increased by 3.4% and 7.1% respectively. This fully demonstrates that each module plays a significant and irreplaceable positive role in enhancing leakage rate prediction performance: CNN effectively extracts local spatio-temporal features, LSTM captures long-term temporal dependencies, while the attention mechanism further optimizes feature representation by focusing on critical time steps. The integration of these modules also consistently enhances valve fault classification performance. Compared to the LSTM model, the complete model achieves a 35.5% improvement in accuracy and a 51.6% increase in F1-Score. Compared to the CNN-LSTM model, it achieves a 30.4% improvement in accuracy and a 38.8% increase in F1-Score.

In ablation experiments, the performance degradation of two key variant models was particularly pronounced: First, for the model without feature input branching, the regression task RMSE surged from 2.990 to 6.825 mL/s, while R^2^ dropped from 0.978 to 0.911; For the classification task, accuracy dropped from 0.922 to 0.562, and the F1-Score decreased from 0.920 to 0.577. Second, the variant retaining only the fully connected network (i.e., removing the CNN-LSTM-Additive Attention branch) exhibited even more severe performance degradation: the regression task RMSE reached 8.168 mL/s, R^2^ was only 0.880, and MAE hit 6.081 mL/s; For the classification task, accuracy dropped to 0.690 with an F1-Score of 0.642, completely failing to capture deep temporal patterns and local transient features within the signals.

Furthermore, comparing the performance of the full multi-task model with the single-task model reveals that for regression tasks, the full model’s RMSE (2.990 mL/s) is significantly lower than the single-task regression model’s 5.553 mL/s, while its R^2^ (0.978) exceeds the single-task model’s 0.946. For classification tasks, the complete model’s accuracy (0.922) and F1-Score (0.920) significantly outperformed the single-task classification model’s 0.831 and 0.801. This demonstrates that the multi-task learning framework enables information exchange and performance complementarity between regression and classification tasks. Compared to single-task learning, it achieves superior performance on both tasks, fully validating the superiority of the multi-task deep learning fusion architecture when simultaneously handling leakage rate prediction and valve fault type identification.

**Figure 14:**
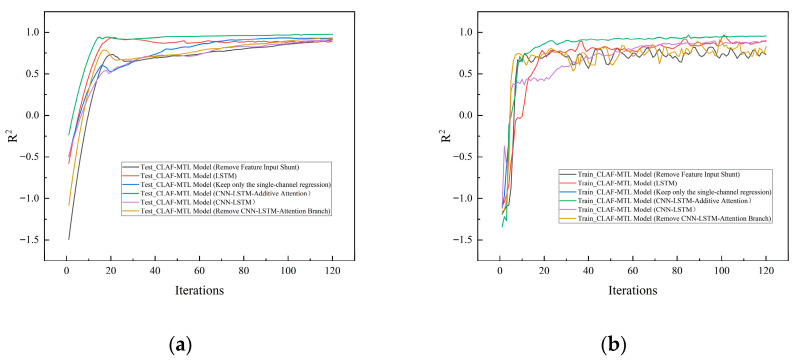
R^2^ Convergence Curves for Training and Test Sets of Various Melting Models. (**a**) R^2^ convergence curve for the test set; (**b**) R^2^ convergence curve for the training set.

The R^2^ convergence curves for the training and test sets of each ablation model are shown in Figure 14. The R^2^ convergence curves reveal that the full model exhibits rapid convergence and strong stability: after 20 iterations, both the training and test set R^2^ values exceeded 0.95, ultimately stabilizing around 0.978. The high alignment between training and testing curves indicates no significant overfitting. This performance demonstrates that the hybrid architecture “CNN-LSTM-Additive Attention + Multi-domain Feature FCN” efficiently captures core features in leakage signals while effectively avoiding overfitting to noise information, laying the foundation for high model performance. The absence of key branches leads to imbalanced model convergence: after removing the feature input branch, the test set R^2^ convergence value drops to only 0.911, with multiple fluctuations occurring during iteration. This indicates that domain-specific prior knowledge provided by multi-domain manual features effectively constrains the model training direction, preventing deep networks from deviating from essential fault characteristics due to a lack of physically meaningful guidance. Conversely, removing the CNN-LSTM-Attention branch resulted in a convergence curve persistently remaining at a low level. This further validates the irreplaceable role of deep auto-features in capturing complex nonlinear leakage evolution patterns. The synergistic effect of these two branches is crucial for ensuring stable model convergence.

**Figure 15:**
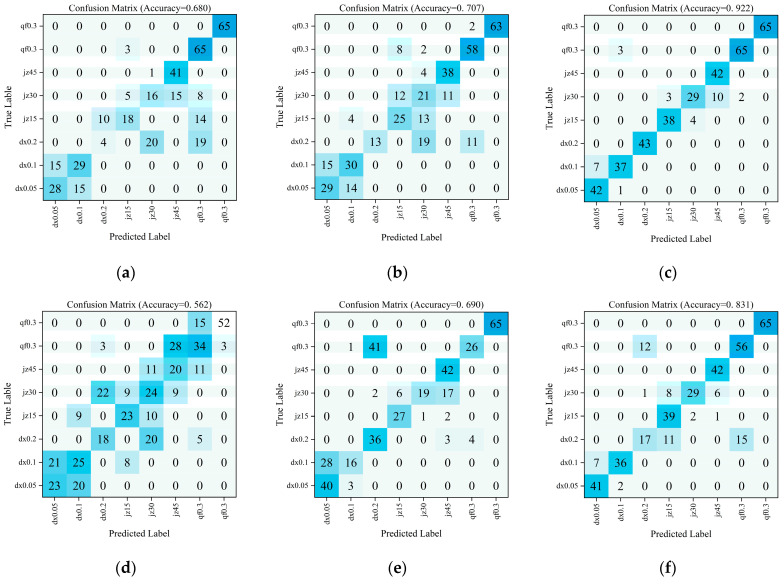
Confusion Matrix for Predicting Failure Types Across Various Ablation Models. (**a**) CLAF-MTL Model (LSTM); (**b**) CLAF-MTL Model (CNN-LSTM); (**c**) CLAF-MTL Model (CNN-LSTM-Additive Attention); (**d**) CLAF-MTL Model (Remove Feature Input Shunt); (**e**) CLAF-MTL model (Remove CNN-LSTM-Attention Branch); (**f**) CLAF-MTL model (Keep only the single-channel regression).

The confusion matrices for fault type prediction across model variants are shown in Figure 15, presenting the analysis results for six ablation experiment models. The complete CLAF-MTL model confusion matrix exhibits concentrated sample distributions across categories with minimal misclassification points, achieving the highest classification accuracy of 92.2%. In contrast, variants lacking critical modules not only show a significant overall accuracy decline but also exhibit pronounced misclassification within the same valve type and cross-misclassification between different valve types.

In summary, ablation experiments systematically validated the effectiveness and necessity of each component in the proposed model for dual-task performance, fully demonstrating the superiority of the multi-task deep learning fusion architecture. The integration of the CNN-LSTM-Additive Attention architecture, multi-domain feature fully connected network architecture, and multi-task learning framework is crucial for achieving high-precision leakage rate prediction and valve failure type identification.

### 5.3. Effectiveness Analysis of the MGDA-UB Algorithm

**Figure 16:**
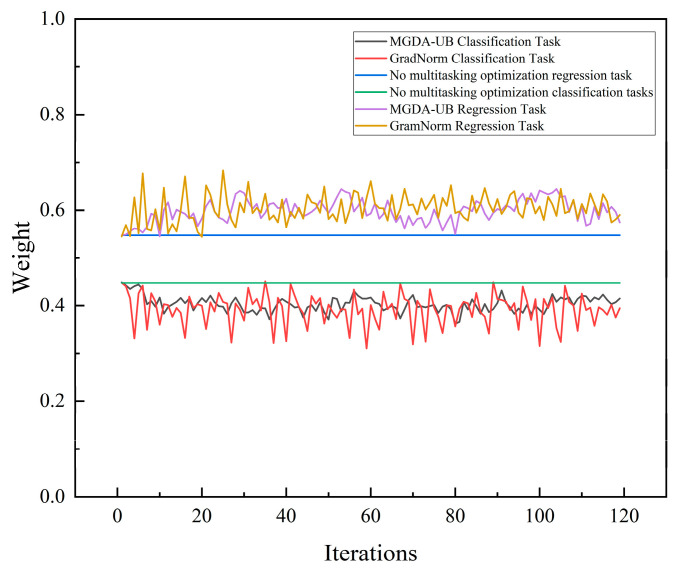
Weight Change Curves for Various Multi-Task Optimization Algorithms.

To validate the adaptability of the MGDA-UB algorithm in the dual-task of hydraulic valve leakage diagnosis, the classic multi-task optimization algorithm GradNorm and a non-multi-task optimization (fixed-weight) approach were selected as comparators. The initial weight configuration was set to 1.0 for the regression task and 0.9 for the classification task. After normalization, the regression task weight became 0.526 and the classification task weight became 0.474. The weight changes for each algorithm are shown in Figure 16.

Due to the relatively stable weight curve of the MGDA-UB algorithm, which stems from its reliance on achieving dual-task coordination through the Pareto optimal common direction in the gradient space rather than adjusting loss weights, while the GradNorm algorithm requires continuous dynamic adjustment of task loss weights to match the logical correspondence of gradient norms across tasks, the results in Figure 16 show that the MGDA-UB algorithm exhibits a relatively stable weight curve. While the GradNorm algorithm exhibits frequent and significant fluctuations in its weight curve.

The dual-task performance metrics for the three algorithms are summarized in Table 5. A variant excluding the multi-task optimization algorithm (fixed weights) is included for reference, providing an intuitive comparison of the algorithms’ optimization value and performance differences:

Analysis of the results in the table above shows that MGDA-UB achieves the highest R^2^ (0.978) and the lowest RMSE (2.990 mL/s) and MAE (2.037 mL/s). GradNorm (R^2^ = 0.964) exhibits significantly higher errors compared to the unoptimized approach. Concurrently, MGDA-UB demonstrates optimal accuracy (0.922) and F1-Score (0.920), while the classification performance of GradNorm and the unoptimized approach progressively deteriorates.

**Figure 17:**
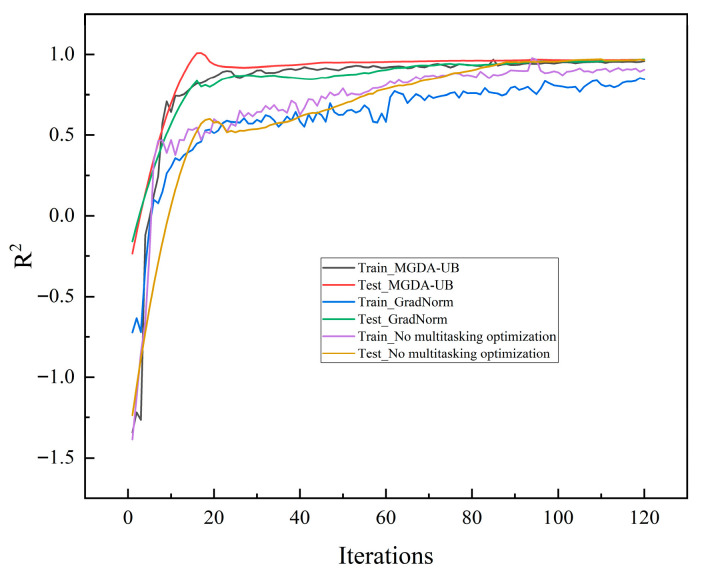
R^2^ Convergence Curves for Training and Test Sets of Various Multi-Task Optimization Algorithms.

As shown in Figure 17, in terms of convergence speed, the training and testing curves of MGDA-UB rapidly climb above 0.5 within 20 iterations, significantly faster than GradNorm and the non-multi-task optimization approach. Notably, the training curve without multi-task optimization exhibits severe fluctuations in the early stages, reflecting the disruption caused by dual-task gradient conflicts during training under fixed weights. Regarding convergence stability, the training and testing curves of MGDA-UB closely align in the later stages with minimal fluctuations, ultimately stabilizing near 0.978. In contrast, GradNorm exhibits divergence between its training and testing curves, while the curve without multi-task optimization shows even more pronounced fluctuations. These patterns further validate the optimization advantages of the MGDA-UB algorithm.

**Figure 18:**
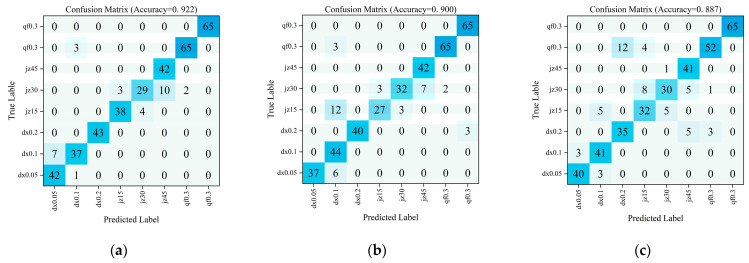
Confusion matrices for various multi-task optimization algorithms (**a**) MGDA-UB algorithm; (**b**) GradNorm algorithm; (**c**) No multi-task optimization.

Figure 18 presents the confusion matrix results of three different multi-task optimization schemes on the test set samples. The classification accuracy rates for each scheme across valve internal leakage fault types are as follows: MGDA-UB algorithm at 92.2%; GradNorm algorithm at 90.0%; and the scheme without multi-task optimization at 88.7%. The approach without multi-task optimization exhibits the lowest classification accuracy, with numerous misclassifications occurring between leakage severity types of similar levels. While the GradNorm algorithm outperforms the non-multi-task optimization approach, its misclassification rate remains higher than that of the MGDA-UB algorithm. Therefore, in the multi-task task of predicting generic valve leakage rates and identifying fault types, the MGDA-UB algorithm demonstrates superior performance compared to both the GradNorm algorithm and the approach without multi-task optimization.

In summary, MGDA-UB is better suited for this research scenario. On one hand, it breaks free from the constraints of loss metrics, effectively addressing the imbalance in gradient adjustment caused by significant differences in loss metrics between the dual tasks of leakage rate regression and fault type classification. On the other hand, it computes gradient direction consistency solely for shared layer parameters, substantially reducing redundant operations in gradient computation and lowering computational complexity. This adaptation to high-dimensional input scenarios enables more stable support for dual-task collaborative optimization, demonstrating superior performance and stability compared to GradNorm and non-multi-task optimization approaches.

## 6. Discussion of Limitations

(1) This study focuses on leak diagnosis for three typical industrial valve types: check valves, globe valves, and solenoid ball valves. The experimental conditions are fixed at 6.0 MPa, but its applicability is constrained by valve type coverage, operating conditions, and modal information limitations, presenting certain limitations in actual industrial scenarios. The model has not been validated for special-function valves such as pressure-reducing valves and relief valves. The leakage mechanisms of these valves differ significantly from those of mechanical failure-type leaks, making direct transfer prone to insufficient feature adaptation. Experiments did not cover extreme conditions like ultra-high pressure (≥ 10 MPa) or temperatures below −20 °C or above 80 °C, which can cause nonlinear signal distortion and weaken feature capture capabilities. Furthermore, reliance solely on vibration and pressure bimodal data, without integrating more sensitive information like acoustic emission or temperature for micro-leaks, may reduce diagnostic accuracy for extremely low leakage rates. Future research will expand coverage of valve types and operating conditions by constructing a comprehensive dataset incorporating special valve configurations and extreme scenarios. Integrating acoustic emission and temperature sensors will establish a multimodal acquisition system. Designing an adaptive modal fusion mechanism based on attention mechanisms will dynamically adjust signal weights across modalities, enhancing the model’s environmental adaptability and micro-leakage diagnostic capabilities.
(2) Although the proposed CLAF-MTL model effectively combines qualitative classification with quantitative prediction, limitations remain in terms of model interpretability, data robustness, and practicality for industrial deployment. Regarding interpretability, while incorporating physical prior knowledge from multi-domain handcrafted features, the deep temporal features processed by the CNN-LSTM-Additive Attention module lack explicit quantitative mapping to failure mechanisms such as valve core eccentricity. This limitation confines the model to feature clustering, rendering it inadequate for industrial fault root cause analysis and maintenance decision-making. Data robustness is compromised as the training set predominantly covers typical failures, with insufficient representation of rare faults like valve core corrosion. For industrial deployment, the model’s large parameter count fails to meet real-time inference demands on edge devices. Future work will enhance model interpretability through techniques like Grad-CAM and feature attribution analysis to establish quantitative mappings between deep features and physical fault parameters. Transfer learning and few-shot learning will improve rare fault diagnosis capabilities, while introducing label noise robust loss functions will mitigate the impact of low-quality data. Model lightweighting via pruning and knowledge distillation will enhance industrial applicability and deployment feasibility.

## 7. Conclusions

This paper addresses bottlenecks in hydraulic valve leakage fault diagnosis, such as poor cross-valve-type universality and the separation of quantitative and qualitative tasks. It innovatively proposes a feature deep fusion-based multi-task learning model (CLAF-MTL) that simultaneously achieves high-precision regression of hydraulic valve leakage rates and accurate classification of fault types. The main innovations and contributions are as follows:

(1) The CNN-LSTM-Attention module automatically extracts deep temporal dynamic features from signals. Combined with multi-domain manual features from time, frequency, and wavelet domains, it forms a fusion feature representation with physical interpretability and high discriminative power. Dimension reduction results via PCA and t-SNE reveal that the fusion features exhibit a clear hierarchical structure in feature space: “valve-type major clusters + operating condition parameter subclusters” This structure markedly outperforms the overlapping and scattered patterns observed with either single automatic features or manual features alone, effectively overcoming the limitations of insufficient expressive power in single-modality feature representations.
(2) To address the practical needs of hydraulic valve leakage diagnosis, this study integrates leakage rate regression and fault type classification into a unified modeling framework. By introducing the MGDA-UB algorithm for dynamic optimization of dual-task weights, it achieves complementary information exchange and collaborative optimization between tasks. Experimental results demonstrate that this framework achieves an R^2^ of 0.978 for the regression task and an accuracy rate of 92.23% for the classification task on the test set. Its performance significantly outperforms single-task models (regression single-task R^2^ = 0.946, classification single-task accuracy = 83.1%), markedly enhancing the model’s diagnostic capability and robustness across diverse valve scenarios.
(3) Covering three typical valve types—check valves, globe valves, and solenoid ball valves—this solution addresses the challenge of feature adaptation across diverse valve structures and failure mechanisms through multi-sensor signal fusion. It delivers an integrated intelligent operation and maintenance solution for complex industrial piping systems, encompassing non-destructive testing, fault identification, and leakage quantification.

The proposed method provides a viable approach for multi-task fault diagnosis of generic valves, offering important practical insights for developing non-destructive testing techniques for internal leakage in such valves.

## Figures and Tables

**Table 1:** Valve Type Designations, Failure Categories, Damage Severity Levels, and Naming Conventions.

Marking	Valve Type	Degree of Injury	Naming
1	Check valve	Eccentricity: 0.05 mm	dx0.05
2	Check valve	Eccentricity: 0.1 mm	dx0.1
3	Check valve	Eccentricity: 0.2 mm	dx0.2
4	Check valve	Normal	dx
5	Shut-off valve	Opening 5% (15°)	jz15
6	Shut-off valve	Opening 10% (30°)	Jz30
7	Shut-off valve	Opening 15% (45°)	Jz45
8	Shut-off valve	Normal	jz
9	Electromagnetic Ball Valve	Valve core return obstruction: 0.3 mm	qf0.3
10	Electromagnetic Ball Valve	Valve core return obstruction: 0.32 mm	qf0.32
11	Electromagnetic Ball Valve	Normal	qf

**Table 2:** Serial numbers, models, parameters, and measurement ranges of various sensors.

Number	Model	Sensitivity\mV/g	Range
1	1A111E	98.196	50 g
2	1A111E	100.254	
3	1A314E	99.666	
		101.332	
		97.941	
4	1A314E	101.430	
		102.704	
		98.098	

**Table 3:** Multi-Task Model Prediction Accuracy Evaluation Results.

Evaluation Dimensions	Evaluation Indicators	Overall	Check Valve	Shut-Off Valve	Electromagnetic Ball Valve
Leakage Rate Prediction	RMSE (mL/s)	2.990	3.066	3.779	1.622
	MAE (mL/s)	2.037	2.721	2.262	0.942
	R^2^	0.978	0.966	0.921	0.982
Valve Failure Type Identification	Accuracy rate	0.922	0.898	0.892	0.965
	Precision	0.910	0.920	0.836	0.960
	Recall rate	0.920	0.930	0.790	0.980
	F1-Score	0.920	0.897	0.827	0.975

**Table 4:** Comparison of Melting Experiment Results.

Model Variants	Return Mission			Classification Task		
	RMSE(mL/s)	MAE(mL/s)	R^2^	Accuracy	Precision	F1-Score
CLAF-MTL Model (LSTM)	7.342	5.661	0.883	0.680	0.654	0.620
CLAF-MTL Model (CNN-LSTM)	6.369	4.203	0.913	0.707	0.693	0.663
CLAF-MTL Model (CNN-LSTM-Additive Attention)	2.990	2.037	0.978	0.922	0.910	0.920
CLAF-MTL Model (Remove Feature Input Shunt)	6.825	4.243	0.911	0.562	0.523	0.577
CLAF-MTL Model (Remove CNN-LSTM-Attention Branch)	8.168	6.081	0.880	0.690	0.673	0.642
CLAF-MTL Model (Keep only single-line classification)	/	/	/	0.831	0.813	0.801
CLAF-MTL Model (Keep only the single-channel regression)	5.553	3.801	0.946	/	/	/

**Table 5:** Comparison Table of Dual-Task Performance for Various Multi-Task Optimization Algorithms.

Algorithm Type	Return Mission			Classification Task		
	RMSE(mL/s)	MAE(mL/s)	R^2^	Accuracy	Precision	F1-Score
MGDA-UB	2.990	2.037	0.978	0.922	0.910	0.920
GradNorm	3.853	3.081	0.964	0.900	0.909	0.890
No multitasking optimization	3.795	3.012	0.965	0.887	0.846	0.867

## Data Availability

Expressions of gratitude are extended to Kaitian New Agricultural Science and Technology Co. in Hunan Province, China, for their provision of the designated test site, and to Hu Haoyu and Li Mi for their invaluable contributions to the present paper.
